# Genomic surveillance of methicillin-resistant *Staphylococcus aureus* in the Philippines,  2013–2014

**DOI:** 10.5365/wpsar.2020.11.1.004

**Published:** 2021-02-26

**Authors:** Melissa L. Masim, Silvia Argimón, Holly O. Espiritu, Mariane A. Magbanua, Marietta L. Lagrada, Agnettah M. Olorosa, Victoria Cohen, June M. Gayeta, Benjamin Jeffrey, Khalil Abudahab, Charmian M. Hufano, Sonia B. Sia, Matthew T.G. Holden, John Stelling, David M. Aanensen, Celia C. Carlos, on behalf of the Philippines Antimicrobial Resistance Surveillance Program

**Affiliations:** a Antimicrobial Resistance Surveillance Reference Laboratory, Research Institute for Tropical Medicine, Muntinlupa, Philippines.; b Centre for Genomic Pathogen Surveillance, Wellcome Genome Campus, Hinxton, England, United Kingdom of Great Britain and Northern Ireland.; c University of St Andrews School of Medicine, St Andrews, Scotland, United Kingdom of Great Britain and Northern Ireland.; d Brigham and Women’s Hospital, Boston (MA), United States of America.; e Centre for Genomic Pathogen Surveillance, Big Data Institute, University of Oxford, Oxford, England, United Kingdom of Great Britain and Northern Ireland.; † These authors contributed equally to this work.; * These authors contributed equally to this work.

## Abstract

Methicillin-resistant *Staphylococcus aureus* (MRSA) remains one of the leading causes of both nosocomial and community infections worldwide. In the Philippines, MRSA rates have remained above 50% since 2010, but resistance to other antibiotics, including vancomycin, is low. The MRSA burden can be partially attributed to pathogen-specific characteristics of the circulating clones, but little was known about the *S. aureus* clones circulating in the Philippines.

We sequenced the whole genomes of 116 *S. aureus* isolates collected in 2013–2014 within the Antimicrobial Resistance Surveillance Program. The multilocus sequence type, *spa* type, SCC*mec* type, presence of antimicrobial resistance (AMR) determinants and virulence genes and relatedness between the isolates were all derived from the sequence data. The concordance between phenotypic and genotypic resistance was also determined.

The MRSA population in the Philippines comprised a limited number of genetic clones, including several international epidemic clones, such as CC30-*spa*-t019-SCC*mec*-IV-PVL+, CC5-SCC*mec*-typeIV and ST239-*spa*-t030-SCC*mec*-typeIII. The CC30 genomes were related to the South-West Pacific clone but formed a distinct, diverse lineage, with evidence of global dissemination. We showed independent acquisition of resistance to sulfamethoxazole/trimethoprim in various locations and genetic clones but mostly in paediatric patients with invasive infections. The concordance between phenotypic and genotypic resistance was 99.68% overall for eight antibiotics in seven classes.

We have made the first comprehensive genomic survey of S. aureus in the Philippines, which bridges the gap in genomic data from the Western Pacific Region and will constitute the genetic background for contextualizing prospective surveillance.

Methicillin-resistant *Staphylococcus aureus* (MRSA) remains one of the leading causes of both nosocomial and community infections worldwide. (*1*) Asian countries such as China, Japan, the Republic of Korea and China, Taiwan (China) have reported high prevalence rates of 70–80% for nosocomial MRSA. (*2*, *3*) In the Philippines, the MRSA rates have increased steadily since 2004 and remained above 50% since 2010, while resistance rates to antibiotics other than β-lactams are low (*4*, *5*) (**Fig. 1A-B**).

**Figure 1:**
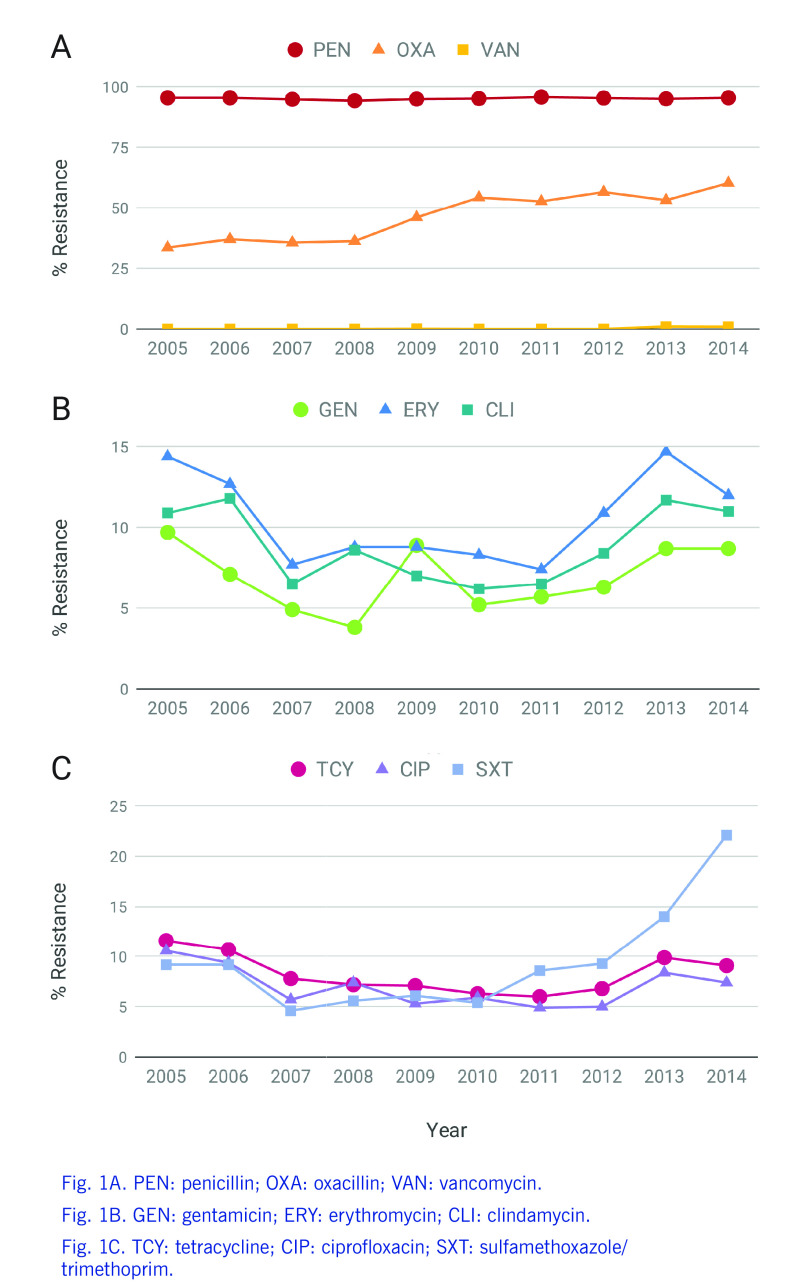
Annual resistance rates of S. aureus isolates to nine antibiotics, 2005–2014

[**insert** Figure 1]

Several notable epidemic clones have spread across Asia, their multilocus sequence types (MLSTs) being ST30 (China, Hong Kong Special Administrative Region SAR [China], Japan, Kuwait, Malaysia, the Philippines, Singapore and China, Taiwan [China]), ST239 (China, India, the Philippines, the Republic of Korea, China, Taiwan [China], Thailand and Viet Nam), ST5 (China, Hong Kong Special Administrative Region SAR [China], Japan, the Philippines, the Republic of Korea, Sri Lanka and China, Taiwan [China]), ST59 (China, Hong Kong Special Administrative Region SAR [China], Sri Lanka, China, Taiwan [China] and Viet Nam) and ST72 (Republic of Korea). (*3*, *6*, *7*) MRSA strains have emerged independently in the context of different epidemic clones (*8*) by acquiring the staphylococcal cassette chromosome *mec* (SCC*mec*) that carries the *mec*A or *mec*C gene, which confers resistance to methicillin and most β-lactam antibiotics. Importantly, some MRSA clones have also acquired resistance to vancomycin, the first-line antibiotic treatment for severe MRSA infections in hospitals, (*9*) although vancomycin resistance has remained very low in the Philippines. (*5*)

Current infection control in the Philippines includes following patients with MRSA infection and laboratory-based surveillance to determine the antimicrobial susceptibility pattern. The MRSA burden can, however, be attributed partially to pathogen-specific characteristics of the circulating clones, such as antibiotic resistance and virulence genes. (*1*) Hence, good understanding of the genomic epidemiology of MRSA in the Philippines will aid in the control and management of MRSA infections.

## Methods

### Bacterial isolates

Data on a total of 6211 *S. aureus* isolates were collected by the Antimicrobial Resistance Surveillance Program (ARSP) of the Philippines Department of Health during the period January 2013 to December 2014. Isolates found to be resistant to oxacillin (i.e. MRSA) were subsequently referred to the ARSP reference laboratory for confirmation. Of the 412 and 384 isolates referred in 2013 and 2014, respectively, a total of 118 MRSA isolates from 17 sentinel sites were selected for whole-genome sequencing (WGS) on the basis of their resistance profile  (**Table 1**), with the following criteria: i) referred to the ARSP reference laboratory in 2013–2014; ii) complete resistance profile (i.e. no missing susceptibility data); iii) overall prevalence of the resistance profile in the ARSP data (both referred and non-referred isolates); iv) geographical representation of different sentinel sites, with the number of isolates included from each site proportional to their relative abundance and estimated from (n/N)*100 (rounded up), where n is the total number of isolates from one site and N is the grand total of isolates; and v) when both invasive and non-invasive isolates representing a combination of resistance profile, sentinel site and year of collection were available, invasive isolates (i.e. from blood, or cerebrospinal, joint, pleural and pericardial fluids) were given priority. We used a proxy definition for “infection origin,” whereby the first isolates collected from patients in the community or on either of the first two days of hospitalization were categorized as isolates from community-acquired infections, while isolates collected on day three in hospital or later were categorized as isolates from hospital-acquired infections. (*10*)

**Table 1:** Numbers of *S. aureus* and MRSA isolates analysed by the ARSP and referred to the reference laboratory during 2013 and 2014, isolates submitted for WGS and high-quality MRSA genomes obtained, discriminated by sentinel site and AMR profile

-	Number of isolates		
	2013	2014	Total
**Total ARSP**			
*S. aureus*	2682	3529	6211
MRSA	1421 (53%)	2128 (60%)	3549 (57%)
**Referred to reference laboratory**			
*S. aureus*	412	384	796
MRSA	381 (92%)	354 (92%)	735 (92%)
**MRSA submitted for WGS**	57	61	118
**MRSA high-quality genomes**	55	61	116
*By sentinel site*^a^			
**BGH**	6	3	9
**BRH**	0	1	1
**CMC**	2	2	4
**CVM**	6	4	10
**DMC**	4	3	7
**EVR**	2	3	5
**FEU**	3	3	6
**GMH**	2	3	5
**JLM**	2	4	6
**MAR**	4	5	9
**MMH**	1	2	3
**NKI**	3	4	7
**NMC**	3	3	6
**SLH**	0	1	1
**STU**	5	5	10
**VSM**	12	13	25
**ZMC**	0	2	2
*By AMR profile*			
PEN OXA	45	59	104
PEN OXA SXT	7	2	9
PEN OXA GEN ERY CLI TCY CIP	2	0	2
PEN OXA ERY	1	0	1

^a^**: BGH: Baguio General Hospital and Medical Center; BRH: Batangas Medical Center; CMC: Cotabato Regional and Medical Center; CVM: Cagayan Valley Medical Center; DMC: Southern Philippines Medical Center; EVR: Eastern Visayas Regional Medical Center; FEU: Far Eastern University – Nicanor Reyes Medical Foundation; GMH: Governor Celestino Gallares Memorial Hospital; JLM: Jose B. Lingad Memorial Regional Hospital; MAR: Mariano Marcos Memorial Hospital & Medical Center; MMH: Corazon Locsin Montelibano Memorial Regional Hospital; NKI: National Kidney and Transplant Institute; NMC: Northern Mindanao Medical Center; SLH: San Lazaro Hospital; STU: University of Santo Tomas Hospital; VSM: Vicente Sotto Memorial Medical Center; ZMC: Zamboanga City Medical Center.**

b CIP: ciprofloxacin; CLI: clindamycin; ERY: erythromycin; GEN: gentamycin; PEN: penicillin; OXA: oxacillin; SXT: sulfamethoxazole/trimethoprim; TCY: tetracycline.

### Antimicrobial susceptibility testing

All *S. aureus* isolates in this study were tested for susceptibility to 14 antibiotics in eight classes: penicillin, oxacillin, cefoxitin, chloramphenicol, sulfamethoxazole/trimethoprim, gentamicin, erythromycin, clindamycin, tetracycline, ciprofloxacin, levofloxacin, rifampicin, linezolid and vancomycin. The susceptibility of the isolates was determined at the ARSP reference laboratory with the Kirby-Bauer disc diffusion method and/or a Vitek 2 Compact automated system (bioMérieux, Marcy-l’Étoile, France). The zone of inhibition and the minimum inhibitory concentration of antibiotics were interpreted according to the 26th edition of the Clinical and Laboratory Standard Institute guidelines. (*11*)

### DNA extraction and whole-genome sequencing

A total of 118 MRSA isolates were shipped to the Wellcome Trust Sanger Institute for WGS. DNA was extracted from a single colony of each isolate with a QIAamp 96 DNA QIAcube HT kit and QIAcube HT (Qiagen, Hilden, Germany). DNA extracts were multiplexed and sequenced on the Illumina HiSeq platform (Illumina, CA, USA) with 100-bp paired-end reads. Raw sequence data were deposited in the European Nucleotide Archive under study accession No. PRJEB17615. Run accessions are provided on the Microreact projects.

### Bioinformatics analysis

Genome quality was evaluated with metrics generated from raw read files, assembly files, annotation files and alignment of the isolates to the reference genome of *S. aureus* subsp. *aureus* strain TW20 (accession FN433596), as previously described. (*12*) Annotated assemblies were produced as previously described. (*13*) Briefly, sequence reads were assembled with VelvetOptimiser v2.2.5 and Velvet v1.2. Automated annotation was performed with PROKKA v1.5 and a genus-specific database from RefSeq. A total of 116 high-quality *S. aureus* genomes were included in the study, characterized by assemblies of < 60 contigs and N50 > 144 000.

We derived the MLST, the *spa* type and SCC*mec* type of the isolates in silico from the whole-genome sequences. The sequence types (STs) were determined from assemblies with Pathogenwatch (https://pathogen.watch/) or from sequence reads with ARIBA (*14*) and the *S. aureus* database hosted at PubMLST. (*15*) The *spa* type was inferred with *spa*Typer v1.0. (*16*) The SCC*mec* type was derived from sequence reads with SRST2 (*17*) and the database available at http://www.sccmec.org/joomla3/index.php/en/.

Evolutionary relations among isolates were inferred from single nucleotide polymorphisms (SNPs) by mapping the paired-end reads to the reference genomes of *S. aureus* strain TW20 (FN433596) or ILRI_Eymole1/1 (NZ_LN626917) with the Burrows Wheeler aligner (BWA) v0.7.12, as described in detail previously. (*12*) Mobile genetic elements were masked in the alignment of pseudogenomes with a script available at https://github.com/sanger-pathogens/remove_blocks_from_aln. For clonal complex (CC) 30 phylogeny, recombination regions detected with Gubbins (*18*) were also removed. SNPs were extracted with snp_sites, (*19*) and maximum likelihood phylogenetic trees were generated with RAxML (*20*) and the generalized time-reversible model with the GAMMA method of correction for among-site rate variation and 100 bootstrap replications. The tree of 7821 global *S. aureus* genomes available at the European Nucleotide Archive with geolocation and isolation date was inferred by an approximately maximum likelihood phylogenetic method with FastTree. (*21*) The CC30 genomes were contextualized with global genomes by using Pathogenwatch (https://pathogen.watch/), which infers trees based on genetic similarity and predicts genotypic AMR. Genome assemblies were generated from read files as described above or downloaded from the National Center for Biotechnology Information if raw Illumina data were not made available. The project and sample accessions are listed on the Pathogenwatch table (https://pathogen.watch/collection/vi3stmhtqnbs-arsp-sau-cc30-2013-2014-global).

Known AMR determinants and the Panton-Valentine leukocidin (PVL) *lukF-PV* and *lukS-PV* genes were identified from raw sequence reads with ARIBA (*14*) and a curated database of known resistance genes and mutations. (*22*) Resistance was predicted from the presence of known AMR genes and mutations identified in the genome sequences. The genomic predictions of AMR (test) were compared with the phenotypic results (reference), and the concordance between the two methods was computed for each of eight antibiotics (928 total comparisons). Isolates with either a resistant or an intermediate phenotype were considered non-susceptible for comparison purposes. An isolate with the same outcome for both the test and reference (i.e. both susceptible or both non-susceptible) was counted as a concordant isolate. The concordance was the number of concordant isolates over the total number of isolates assessed (expressed as per cent).

All project data, including inferred phylogeny, AMR predictions and metadata, were made available through the web application Microreact (http://microreact.org).

### Ethics statement

Ethical approval is not applicable. This study is based on archived bacterial samples processed by ARSP. No identifiable data were used in this study.

## Results

### Demographics and characteristics of MRSA isolates

Of the 118 MRSA isolates submitted for WGS, 116 were confirmed as *S. aureus* in silico, while two isolates were identified as *Staphylococcus argenteus* and were not included in the downstream analyses. The age range of the patients was < 1 to 90 years; 20% (*n* = 23) of the isolates were from patients aged < 1 year (**Table 2**). Of the 116 isolates, 56% were recovered from male patients (*n* = 66) and 44% from females (*n* = 50). As invasive isolates were prioritized, the most common specimen source was blood (62%, *n* = 72), followed by wounds (19%, *n* = 22). The majority of the infections (68%,  *n* = 79) were classified as community-associated MRSA.

**Table 2:** Demographics and clinical characteristics of 116 MRSA isolates

Characteristic	No. of isolates
**Sex**	
Male	66
Female	50
**Age (years)**	
< 1	23
1–4	9
5–14	16
15–24	14
25–34	8
35–44	17
45–54	14
55–64	9
65–80	1
^3^81	5
**Patient type**	
Inpatient	104
Outpatient	12
**Specimen origin**	
Community-acquired	79
Hospital-acquired	37
**Specimen type**	
Abdominal fluid*	1
Abscess	4
Aspirate	2
Blood*	72
Bone	1
Cerebrospinal fluid*	2
Pericardial fluid*	1
Tracheal aspirate	5
Urine	2
Wound	22
Others	1

Isolates considered to be invasive are those obtained from specimen types marked with an asterisk (*).

### Concordance between phenotypic and genotypic AMR

Isolates were tested for susceptibility to 14 antibiotics in eight classes. All the isolates were susceptible to vancomycin and linezolid and resistant to penicillin, oxacillin and cefoxitin, consistent with the presence of the *blaZ* and *mecA* genes (**Table 3**). Nine isolates were resistant to cotrimoxazole, which was associated with the presence of the *dfrG* gene. Two isolates were multidrug-resistant and carried genes and mutations for resistance to penicillin (*blaZ, mecA*), oxacillin (*mecA*), cefoxitin (*mecA*), gentamicin (*aacA-aphD*), erythromycin (*ermC, msrA*), clindamycin (*ermC*), tetracycline (*tetM, tetK*), ciprofloxacin and levofloxacin (GyrA_S84L, GyrA_G106D, and GrlA_S80F mutations), chloramphenicol (*catA1*) and rifampicin (*rpoB*_H481N). The *IleS* gene that confers resistance to mupirocin and the *sdrM* gene conferring resistance to norfloxacin were identified in three and 23 isolates, respectively; however, mupirocin was not tested in the laboratory, and norfloxacin was tested only against isolates from urine specimens. Hence, these two antibiotics were not included in the concordance analysis.

**Table 3:** Comparison of genomic predictions of antibiotic resistance with laboratory susceptibility testing at the ARSP reference laboratory

Antibiotic class	Antibiotic	Resistant isolates	False-positive	False-negative	Concordance (%)	Resistance genes/SNPs
Penicillin	Penicillin	116	0	0	100	*blaZ, mecA*
Penicillin	Oxacillin	116	0	2	98.28	*mecA*
Folate pathway antagonist	Sulfamethoxazole/ Trimethoprim	9	1	0	99.14	*dfrG*
Aminoglycoside	Gentamicin	2	0	0	100.00	*aacA_aphD*
Lincosamide	Clindamycin	2	0	0	100	*ermC*
Macrolide	Erythromycin	3	0	0	100	*ermC, msrA*
Tetracycline	Tetracycline	2	0	0	100	*tetM, tetK*
Fluoroquinolone	Ciprofloxacin	2	0	0	100	GyrA_S84L, GyrA_G106D, GrlA_S80F

Comparisons between phenotypic and genotypic data are presented for eight key antibiotics in seven classes (**Table 3**). The overall concordance for the 928 comparisons was 99.68%, and the concordance for individual antibiotics was > 98% in all cases (**Table 3**). The notable exceptions were two false-negative results for oxacillin resistance, i.e. isolates confirmed to be phenotypically resistant but without the *mec*A resistance gene. Conversely, one isolate was falsely predicted to be resistant to sulfamethoxazole/trimethoprim on the basis of the presence of the *dfrG* gene.

### Genotypic findings

#### In silico genotyping

MLST, *spa* type and SCC*mec* type were predicted in silico from the WGS data for the 116 MRSA isolates. A total of 18 STs were identified; 74.1% (*n* = 86) of the isolates belonged to clonal complex (CC) 30, distributed between ST30 (*n* = 81), its single-locus variant ST1456 (*n* = 2) and three ST30 genomes showing novel *aroE*  (*n* = 2) and *yqiL* (*n* = 1) alleles. CC5 was represented by nine genomes and ST834 by six. The most prevalent of the 29 different *spa* types identified was t019 (62%), which coincided with genomes assigned to CC30. The *spa* types identified for the CC5 genomes were t002 (*n* = 5), t105 (*n* = 3) and t067 (*n* = 1). Most of the SCC*mec* cassettes identified in the genomes belonged to type IV (*n* = 108, 93.1%), followed by type III  (*n* = 2, 1.7%). The SCC*mec* type could not be determined for four genomes. The numbers and most common ST and *spa* types found in each of the sentinel sites are shown in **Table 4**. Overall, the typing results for the genome sequences showed that CC30-*spa*-t019-SCC*mec*-IV was the most prevalent MRSA clone in this retrospective collection (*n* = 67, 57.8%).

**Table 4:** Distribution of isolates, STs, *spa* types, resistance profiles and AMR genes and mutations at the 17 sentinel sites

Laboratory^b^	No. of isolates	No. of STs	Prevalent ST (no. of isolates)	No. of *spa* types	Prevalent *spa* type (no. of isolates)	Resistance profiles^a^	Resistance genes
BGH	9	4	30 (6)	5	t019 (5)	PEN OXA (9)	*blaZ, mecA* (6) *blaZ, mecA, sdrM* (2) *blaZ, sdrM* (1)
BRH	1	1	30 (1)	1	t019 (1)	PEN OXA (1)	*blaZ, mecA* (1)
CMC	4	1	30 (4)	2	t019 (3)	PEN OXA (4)	*blaZ, mecA* (4)
CVM	10	6	30 (4)	6	t019 (4)	PEN OXA (9) PEN OXA SXT (1)	*blaZ, mecA* (5) *blaZ, mecA, sdrM* (3) *mecA* (1) *blaZ, mecA, dfrG, sdrM* (1)
DMC	7	4	30 (4)	3	t019 (5)	PEN OXA (3) PEN OXA SXT (4)	*blaZ, mecA* (3) *blaZ, mecA, dfrG* (3) *blaZ, mecA, dfrG, sdrM* (1)
EVR	5	3	30 (3)	3	t019 (3)	PEN OXA (5)	*blaZ, mecA* (4) *blaZ, mecA, dfrG, sdrM* (1)
FEU	6	1	30 (6)	3	t019 (3)	PEN OXA (6)	*blaZ, mecA* (6)
GMH	5	1	30 (5)	1	t019 (5)	PEN OXA (5)	*blaZ, mecA* (5)
JLM	6	3	5 (3)	3	t002 (3)	PEN OXA (6)	*blaZ, mecA* (5) *blaZ, mecA, sdrM* (1)
MAR	9	3	30 (6)	2	t019 (7)	PEN OXA (7) PEN OXA GEN ERY CLI TCY CIP (2)	*blaZ, mecA* (7) *blaZ, mecA, aacA_aphD, ermC, tetM, tetK*, GyrA_S84L, GyrA_G106D, GrlA_S80F, *catA1, sdrM, ileS_2* (2)
MMH	3	1	30 (3)	3	t019 (1), t3800 (1), t975 (1)	PEN OXA (3)	*blaZ, mecA* (3)
NKI	7	4	30 (4)	4	t019 (4)	PEN OXA (7)	*blaZ, mecA* (6) *blaZ, sdrM* (1)
NMC	6	1	30 (6)	1	t019 (6)	PEN OXA (6)	*blaZ, mecA* (6)
SLH	1	1	30 (1)	1	t019 (1)	PEN OXA (1)	*blaZ, mecA* (1)
STU	10	3	30 (8)	2	t019 (9)	PEN OXA (10)	*blaZ, mecA* (9) *blaZ, mecA, sdrM* (1)
VSM	25	6	30 (17)	14	t019 (12)	PEN OXA (21) PEN OXA SXT (3) PEN OXA ERY (1)	*blaZ, mecA* (16) *mecA, sdrM* (1) *blaZ, mecA, sdrM* (3) *blaZ, mecA, sdrM, ileS_2* (1) *blaZ, mecA, dfrG* (1) *blaZ, mecA, dfrG, sdrM* (2) *blaZ, mecA, msrA, sdrM* (1)
ZMC	2	2	30 (1), 5 (1)	2	t019 (1), t105 (1)	PEN OXA (1) PEN OXA SXT (1)	*blaZ, mecA* (1) *blaZ, mecA, dfrG, sdrM* (1)

^a^ **PEN: penicillin; OXA: oxacillin; GEN: gentamicin; ERY: erythromycin; CLI: clindamycin; TCY: tetracycline; CIP: ciprofloxacin; SXT: sulfamethoxazole/trimethoprim.**

^b^ BGH: Baguio General Hospital and Medical Center; BRH: Batangas Medical Center; CMC: Cotabato Regional and Medical Center; CVM: Cagayan Valley Medical Center; DMC: Southern Philippines Medical Center; EVR: Eastern Visayas Regional Medical Center; FEU: Far Eastern University – Nicanor Reyes Medical Foundation; GMH: Governor Celestino Gallares Memorial Hospital; JLM: Jose B. Lingad Memorial Regional Hospital; MAR: Mariano Marcos Memorial Hospital & Medical Center; MMH: Corazon Locsin Montelibano Memorial Regional Hospital; NKI: National Kidney and Transplant Institute; NMC: Northern Mindanao Medical Center; SLH: San Lazaro Hospital, STU: University of Santo Tomas Hospital; VSM: Vicente Sotto Memorial Medical Center; ZMC: Zamboanga City Medical Center.

#### Population structure of MRSA in the Philippines

**Figure 2:**
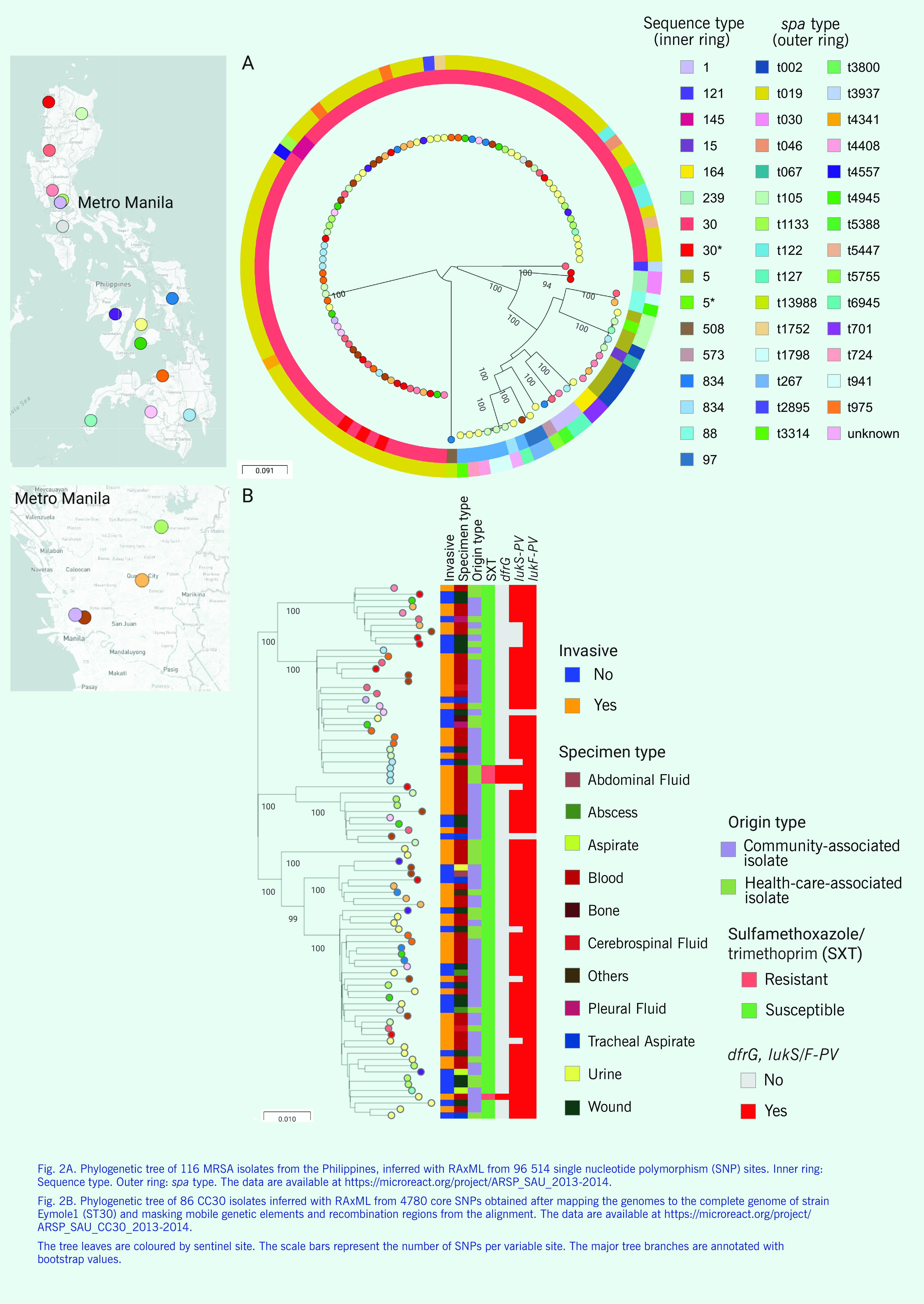
Genomic surveillance of S. aureus from the Philippines, 2013–2014

The phylogenetic tree shows that the population was composed of discrete clades that matched the ST distribution and were separated by long branches  (**Fig. 2A**), in agreement with the clonal population previously described for *S. aureus.* (*23*) The largest clade represented by CC30-*spa*-t019-SCC*mec*-IV was characterized by broad geographical distribution across the 17 sentinel sites in this data set (**Table 4**), as it was found in both community- and health-care-associated isolates obtained from at least 11 different specimen types. WGS revealed distinct major sublineages within the CC30 clade  (**Fig. 2B**), none of which displayed a strong phylogeographical signal. Both genes *lukS-PV* and *lukF-PV* that encode the PVL were found in 75 of the 86 CC30 genomes (**Fig. 2B**), indicating that the majority (87.2%) are PVL-positive (**Fig. 2B**).

[**insert** Figure 2]

Two additional epidemic clones were identified, CC5 and ST239 (CC8). Six of the nine CC5-SCC*mec*-typeIV (71%) were from paediatric patients (compared with 20% of the entire data set) and were generally clustered according to their *spa* type; however, they displayed no clear phylogeographical distribution. Two isolates from different, distant locations carried both *lukS-PV* and *lukF-PV* genes (PVL-positive). The two ST239 isolates were from the same patient, *spa* type t030, SCC*mec*-typeIII, PVL-negative and multidrug-resistant.

The nine isolates with resistance to sulfamethoxazole/trimethoprim were from four locations (Cagayan Valley Medical Center [CVM], Southern Philippines Medical Center [DMC], Vicente Sotto Memorial Medical Center [VSM] and Zamboanga City Medical Center [ZMC]) and belonged to four different clones (CC30, CC5, ST1649 and ST834), which suggests that resistance to this antibiotic has emerged independently (**Table 4**). The isolates were obtained from blood (*n* = 8) and an abscess  (*n* = 1) and, interestingly, mainly from paediatric patients (6 of 7, 85.7% of paediatric patients, in comparison with 46.6% of paediatric patients in the total data set).

#### MRSA in the Philippines in the global context

**Figure 3:**
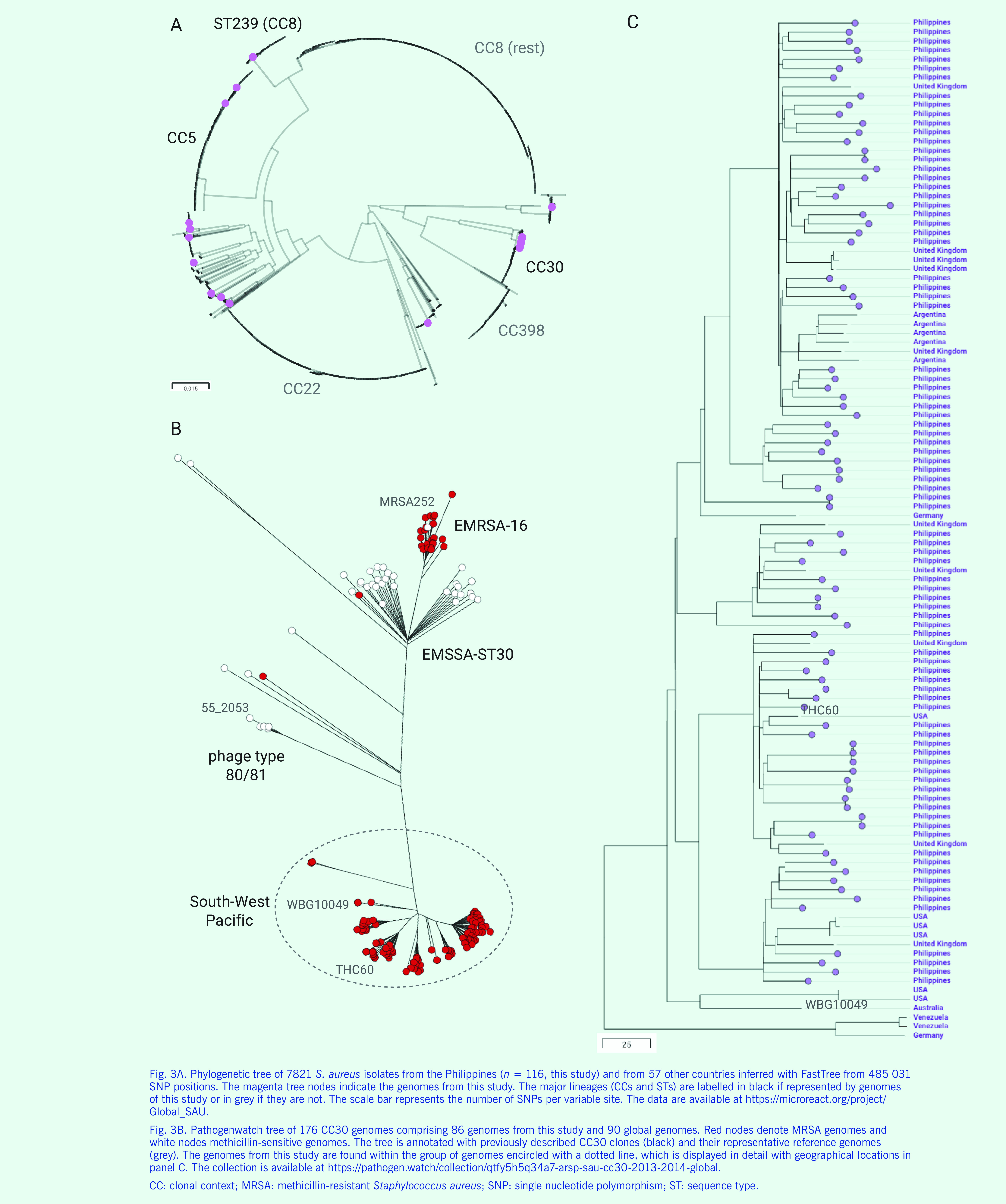
S. aureus from the Philippines in the global context

We placed the genomes from our retrospective collection in the global context of 7821 contemporary *S. aureus* public genomes available from sequence data archives with linked geographical and temporal information, collected between 2010 and 2017. This public collection of genomes represents 57 countries and 379 STs, but it is heavily biased towards genomes from Europe  (*n* = 3556) and the United States of America (*n* = 3241) and the epidemic clones ST8 (*n* = 2343), ST22 (*n* = 1526) and ST5  (*n* = 720) prevalent in those regions (**Fig. 3A**). Health-care-associated EMRSA-15 (ST22) was notably absent from our collection, as was livestock-associated CC398 (**Fig. 3A**). The Philippine ST5 genomes did not form a monophyletic group within the CC5 clade, suggesting more than one origin. CC30-*spa*-t019-SCC*mec*-IV-PVL+ MRSA genomes from the Philippines formed a discrete cluster within ST30 with small numbers of genomes from the United States of America (*n* = 5), the United Kingdom of Great Britain and Northern Ireland (*n* = 3) and Germany (*n* = 1, **Fig. 3B**).

[**insert** Figure 3]

Several successful pandemic clones have emerged within CC30, such as the methicillin-sensitive phage type 80/81, (*24*) the MRSA South-West Pacific clone, (*25*) the hospital-endemic epidemic MRSA-16 (ST36 (*26*)) and epidemic MSSA-ST30. (*27*) We investigated the relations between the Philippine MRSA genomes in this study and these clones with Pathogenwatch. The Philippine genomes were clustered into several clades related to but distinct from the South-West Pacific clone, representing a new diversification from this clone (**Fig. 3B**). In addition, the genomes from the Philippines clustered with genomes from Argentina, Germany, the United Kingdom and the USA (**Fig. 3C**), indicating that the epidemic diversification from the South-West Pacific clone was accompanied by global dissemination.

## Discussion

In this study, we combined WGS and laboratory-based surveillance to characterize MRSA circulating in the Philippines in 2013 and 2014. High levels of concordance between phenotypic and genotypic resistance were observed for all the antibiotics tested. This was previously reported for *S. aureus* collections in Europe and the United Kingdom, (*27*, *28*) but our results, the first from the Philippines, show no significant gaps in the epidemiology of known resistance mechanisms in this country. The integration of laboratory and WGS data showed independent acquisition of resistance to sufamethoxazole/trimethoprim mainly in paediatric patients with invasive infections. This is probably due to the selective pressure of antibiotic use, as co-trimoxazole was recommended by the Department of Health in the Philippines as one of the first-line antibiotics for paediatric patients with pneumonia in the 1990s and is currently the first-line antibiotic for skin and soft tissue MRSA infections in paediatric patients recommended in the Philippines National Antibiotic Guideline for 2017. While none of the isolates referred to the Antimicrobial Resistance Surveillance Reference Laboratory (ARSRL) were confirmed to display intermediate resistance or to be resistant to vancomycin by broth microdilution as per Clinical & Laboratory Standards Institute (CLSI) guidelines, susceptibility testing at ARSRL does not currently include protocols for the detection of vancomycin heteroresistance (hVISA).

Few studies of the molecular epidemiology of MRSA in the Philippines have been published. We found that the MRSA population in 2013 and 2014 comprised a limited number of genetic lineages, dominated by CC30-*spa*-t019-SCC*mec*-IV-PVL+. Community-acquired CC30-*spa*-t019-PVL+ MRSA was previously reported in one hospital in the Philippines between 2004 and 2006 and from other countries in South-East Asia, with potential clonal expansion. (*3*, *6*) This suggests that the increase in the burden of MRSA observed in the Philippines since 2004 is linked to extension of this clone. A longer retrospective sequencing survey would provide more detailed insight into the dynamics of this clone. The lack of a clear phylogeographical structure in this established clone may be related to its ability to disseminate both within hospitals and in the community and within the community, followed by nosocomial transmission, (*29*) in the dense population of the Philippines. However, specimens were collected from patients for clinical purposes, not screening, and only a subset of isolates were sequenced. Thus, we cannot rule out the possibility that the lack of phylogeographic structure is the result of incomplete coverage. In addition, the ARSP hospital-based surveillance would have to be complemented by community surveillance to determine the dynamics of MRSA in the population at large. Only a few publicly available global genomes were found to be closely related to the CC30 genomes in this study, highlighting the paucity of WGS data from continents other than Europe and North America. The availability of other genomes would enhance our understanding of the global epidemiology of this clone.

Genotypic characterization of circulating MRSA strains, with phenotypic and epidemiological data, led to the identification of several global epidemic clones and revealed the lack of a strong phylogeographic structure in the population from patients admitted to health-care facilities in a country with a high burden of MRSA. This supports interventions to reduce the burden of disease in the general population. (*6*) Targeted eradication interventions may be useful in individual hospitals, where high-risk epidemic clones such as ST239 may cause local outbreaks. Our results represent the first comprehensive genomic survey of *S. aureus* in the Philippines, bridging the gap in genomic data from the Western Pacific Region, and provides the genetic background for contextualizing prospective surveillance for infection control.
